# Hurricane Irma’s Impact on Assisted Living Residents’ Rates of Hospitalization, Nursing Home Placement, and Mortality

**DOI:** 10.1093/geroni/igaa057.2610

**Published:** 2020-12-16

**Authors:** Cassandra Hua, Kathryn Hyer, Wenhan Zhang, Jessica Ogarek, David Dosa

**Affiliations:** 1 Brown University School of Public Health, Providence, Rhode Island, United States; 2 University of South Florida, Tampa, Florida, United States; 3 Brown University, Providence, Rhode Island, United States; 4 Brown University, PROVIDENCE, Rhode Island, United States; 5 Brown University, Barrington, Rhode Island, United States

## Abstract

Little is known about the impact of hurricanes on the large and increasingly vulnerable population residing in assisted living communities (ALs). The objective of this paper was to leverage a novel methodology to identify Medicare beneficiaries residing in Florida ALs and determine their outcomes associated with Hurricane Irma in 2017. With Medicare enrollment records, claims, and the nursing home Minimum Data Set, we identified a cohort of AL residents in 2015 (n=45,505) and 2017 (n=42,306) and compared their rates of 30-day hospitalization, nursing home placement, and mortality in the two years. AL residents in 2017 had a 10% increase in their 30-day hospitalization rates (3.96 in 2015, 4.34 in 2017), 16% increase in their 30-day nursing home placement rates (1.61 in 2015, 1.87 in 2017), and 22% increase in their 30-day mortality (0.54 in 2015, 0.66 in 2017). Findings suggest Florida AL residents experienced adverse outcomes following Hurricane Irma.

